# Feeding Conjugated Linoleic Acid without a Combination of Medium-Chain Fatty Acids during Late Gestation and Lactation Improves Pre-Weaning Survival Rates of Gilt and Sow Progeny

**DOI:** 10.3390/ani9020062

**Published:** 2019-02-15

**Authors:** Jessica R. Craig, Frank R. Dunshea, Jeremy J. Cottrell, Erin M. Ford, Udani A. Wijesiriwardana, John R. Pluske

**Affiliations:** 1 Research and Innovation, Rivalea (Australia) Pty. Ltd., Corowa NSW 2646, Australia; eford@rivalea.com.au; 2 Agricultural Sciences, College of Science, Health, Engineering and Education, Murdoch University, Murdoch, WA 6150, Australia; 3 Faculty of Veterinary and Agricultural Sciences, The University of Melbourne, Parkville, VIC 3010, Australia; fdunshea@unimelb.edu.au (F.R.D.); jcottrell@unimelb.edu.au (J.J.C.); udani.wijesiriwardana@unimelb.edu.au (U.A.W.); 4 Australasian Pork Research Institute Ltd. (APRIL), Willaston, SA 5118, Australia; j.pluske@april.org.au

**Keywords:** CLA, conjugated linoleic acid, colostrum, gilt progeny, MCFA, medium-chain fatty acid

## Abstract

**Simple Summary:**

A number of feeding strategies have been used in attempts to improve performance of progeny born to primiparous sows, which are born lighter, grow slower, and have higher rates of mortality than progeny born to older sows. The current study examined whether feeding conjugated linoleic acid (CLA) or a commercial medium-chain fatty acid (MCFA) product alone or in combination to primiparous and multiparous sows improved pre-weaning growth and survival of their progeny. Feeding CLA or MCFA failed to improve reproductive performance of primiparous or multiparous sows or the performance of their progeny during lactation, and there was no added benefit of feeding these products for gilt progeny. However, feeding CLA alone improved the survival of gilt and sow progeny. Further examination of the different inclusion levels and the timing of CLA feeding may be required in order for the use of this additive to be more efficacious.

**Abstract:**

Feeding conjugated linoleic acid (CLA) or medium-chain fatty acids (MCFA) to dams has been shown to improve progeny growth and survival, and hence may be particularly advantageous to gilt progeny. Primiparous (*n* = 129) and multiparous sows (*n* = 123; parities 3 and 4) were fed one of four diets from day 107 of gestation (107.3 ± 0.1 days) until weaning (day 27.2 ± 0.1 of lactation): (i) control diet; (ii) 0.5% CLA diet; (iii) 0.1% MCFA diet; and (iv) equal parts of (ii) and (iii). Progeny performance data were collected and, from a subset of sows (*n* = 78) and their piglets (*n* = 144), a colostrum (day 0), milk (day 21), and piglet serum sample (day 3) were analyzed for immunoglobulin G and several selected metabolites. Liveborn pre-weaning mortality tended to be lowest (*p* = 0.051) in piglets from sows fed 0.5% CLA. However, sows fed the CLA diet had more (*p* = 0.005) stillbirths than those on the other diets. There were few effects of diet or the dam parity x diet interaction (*p ≥* 0.05) on other parameters. Overall, feeding CLA or MCFA did not improve the performance of primiparous sows, multiparous sows, or their progeny.

## 1. Introduction

Feeding higher levels of lipids during late gestation and lactation may be an effective way to improve growth of suckling pigs through increasing energy available in colostrum and milk [1,2,3]. Conjugated linoleic acid (CLA) refers to a group of positional and geometrical isomers of linoleic acid (18:2) that, when fed to the dam in late gestation and (or) lactation, has been shown to alter the fatty acid (FA) profiles of colostrum and milk [4,5,6] and improve the growth performance of progeny [6,7,8,9]. Additionally, piglets born to sows fed CLA have shown improved circulating immunoglobulin G (IgG) concentrations, suggesting a role of CLA for improved passive immunity [4,8,10]. Medium-chain fatty acids (MCFA) are FA that contain six to twelve carbon atoms, possess antibacterial properties, improve intestinal integrity, and can be used as an efficient energy source by enterocytes [11]. Gilt progeny are born lighter [12,13] and may be compromised in terms of passive immunity [14,15] compared to progeny born to multiparous sows. Therefore, feeding CLA or MCFA to primiparous sows might offer improved trans-placental energy transfer and manipulate colostrum and (or) milk nutrient levels in order to improve the pre-weaning performance and survival of gilt progeny. 

Therefore, the aim of the current experiment was to assess the effect of feeding CLA and MCFA, alone or in combination, in late gestation and lactation to primiparous and multiparous sows on the growth and survival of their progeny. We hypothesized that gilt progeny would benefit further from this feeding strategy due to their lower birth weights and reduced absorption of IgG from colostrum in comparison to sow progeny, with improvements in pre-weaning survival due to increased energy stores around birth and higher concentrations of IgG in colostrum and milk, resulting in improved immunity.

## 2. Materials and Methods

### 2.1. Ethics Statement

Experimental procedures were approved by both the Rivalea (Australia) Animal Care and Ethics Committee (protocol number 16P044) and the Murdoch University Animal Ethics Committee (protocol number NS2920/17) according to the Australian Code for the Care and Use of Animals for Scientific Purposes [16].

### 2.2. Experimental Design

A total of 252 first-cross dams (PrimeGro^TM^, Corowa NSW, Australia) were included in the experiment, including 129 primiparous (parity 1) and 123 multiparous sows (parities 3 and 4). Sows were assigned to one of four dietary treatments (Table 1), fed from day 107 of gestation (107.3 ± 0.1 days; mean ± SE) and throughout lactation until weaning (27.2 ± 0.1 days of age). Diets consisted of different sources of dietary lipid: (i) 6% tallow, predominantly ruminant (control; CON); (ii) a CLA diet consisting of 2.5% tallow replaced with a commercial CLA product (Lutrell® Pure; BASF, Southbank Vic, Australia; the product contained 20% CLA isomers comprised of 10% c9,t11-18:2 and 10% t10,c12-18:2); (iii) a MCFA diet consisting of 0.1% tallow replaced with a commercial MCFA product (Aromabiotic® Pig; Nuscience, Drongen, Belgium; comprised of 28.1% acetic acid, 0.9% valeric acid, 0.9% caproic acid, 0.4% caprylic acid, 18.5% capric acid, and 16.5% lauric acid); and (iv) a combination (BOTH) that was delivered to sows as equal parts of the CLA and MCFA diets (by weight) such that sows received 0.25% CLA isomers and 0.025% MCFA product in their diet ration in the place of tallow. The diets were all formulated to be isoenergetic and isonitrogenous, and the analyzed composition of the final diets is shown in Table 2.

Total feed intake was recorded for each sow from farrowing until weaning, and the average daily feed intake (ADFI) in this period was calculated. Dam body weight (BW) and backfat depth measured ultrasonically at the P2 site (6.5 cm from the midline over the last rib) were recorded at entry to the farrowing house. Within 24 h of birth, all live piglets were individually weighed and given a numbered ear tag prior to fostering. Date of birth, number of piglets born alive (NBA), number of stillbirths (SB), number of mummified fetuses (MU), and total piglets born (TB) were recorded for each litter. Piglets were then individually weighed again at day 21 of lactation, and the average daily gain (ADG) in this period for each piglet was calculated. All liveborn piglet mortalities were recorded from birth until weaning, and the number of piglets weaned (NW) was recorded for each litter.

Dam BW and P2 backfat depth were measured again at weaning, and individual BW and P2 backfat changes were calculated. The date and outcome of the subsequent mating and the associated weaning-to-estrus interval (WEI) and gestation length were recorded from the commercial herd data recording system as well as NBA and NW in the subsequent litter. 

### 2.3. Animal Management

The experiment was carried out under commercial conditions at a large piggery in Corowa, New South Wales, Australia (Rivalea Australia Pty. Ltd.). Primiparous and multiparous sows were group-housed separately in groups of 40 (approximately 2 m^2^/pig) from mating and fed a common gestation diet [averaging 13.1 MJ DE/kg, 12.8% CP, 0.5% available standardized ileal digestible (SID) Lys, as fed basis] through to entry to the farrowing house at approximately day 104 (103.9 ± 2.2 days) of gestation. During this period, animals were fed different allowances in early (weeks 1 to 5; 2.4 kg/d and 2.7 kg/d for primiparous and multiparous sows, respectively), mid (weeks 6 to 13; 2.0 kg/d and 2.2 kg/d, respectively), and late gestation (weeks 14 until entry to the farrowing house; 2.2 kg/d and 2.4 kg/d, respectively). Sows were fed the control (CON) diet regardless of their experimental treatment from entry to the farrowing house until day 107 of gestation. Experimental diets were fed from day 107 of gestation. Sows were given an allowance of 2.5 kg/d from entry to the farrowing house until farrowing, 2.5 kg on the day of farrowing, 3 kg on the first day after farrowing, a maximum of 4 kg on the second day and ad libitum access thereafter until weaning. Cross-fostering was carried out 24 hours after farrowing as per commercial procedures to standardize litters to approximately 10 to 12 piglets depending on the number of functional mammary glands. Piglets were fostered within dietary and parity treatments wherever possible.

### 2.4. Sample Collection

Colostrum samples (approximately 5–10 mL) were manually collected at parturition (within 1 hour of the birth of the first piglet) without the use of oxytocin from a subset of sows that farrowed during the day and could therefore be observed at farrowing (“focal sows”, *n* = 78). Milk samples were manually collected on day 21 of lactation after injection of 1 mL (10 IU) of subcutaneous oxytocin (intra-vulval; Ilium Syntocin®, Troy Laboratories, Glendenning NSW, Australia). Colostrum and milk samples were pooled from the first three anterior glands on either side of the udder. Samples were stored immediately at −20 °C until further analysis. Prior to IgG, protein, and lactose analysis, colostrum and milk samples were defatted by centrifuging at 21,000× *g* for 20 min at 4 °C. Due to commercial restraints, piglets were unable to be separated from their dams before blood sample collection. Individual blood samples (approximately 1 mL) were collected into Vacutainer® tubes (BD Australia, North Ryde NSW, Australia) from two piglets per focal sow litter (one male and one female, *n* = 144) via jugular venepuncture 3 days after farrowing. Samples were then left to clot for at least 3 h, centrifuged at 6000× *g* for 5 min, serum aspirated, and stored at −20 °C until further analysis.

### 2.5. Colostrum and Milk Analysis

Colostrum and milk samples were analyzed for total fat, protein, and lactose, from which a final value for total net energy (NE) of milk was calculated using the equation derived by Hansen et al. [17]:

NE (MJ/kg) = 0.389 × Fat (%) + 0.239 × Protein (%) + 0.165 × Lactose (%).

Total NE content of colostrum was derived using the same equation without the value for protein as per the suggestion of Theil et al. [18] that >90% of protein in colostrum is in the form of immunoglobulins, which are not used for energy by the pig per se. Total fat concentration in each whole colostrum and milk sample was measured using a modified version of the method described by Forcato et al. [19]. A pooled colostrum sample collected previously from a number of sows and with a known crude fat concentration determined by Soxhlet extraction [20] at the NSW Department of Primary Industries (Wagga Wagga NSW, Australia) was used to form a standard curve. Sixty μL of sample was added to 3 mL of chilled ethanol (Ajax Finechem; Thermo Fisher Scientific, Scoresby Vic, Australia), shaken, and then frozen at −20 °C for 1 h. The solution was then centrifuged at 13,000× *g* for 15 min at 4 °C, after which 200 μL of the supernatant was added in duplicate to a 96 well UV compatible assay plate (UV-Star® Microplate, Grenier Bio-One; Interpath Services Pty. Ltd., Heidelberg West Vic, Australia) after being left to reach room temperature. The absorbance was then read at 208 nm on a plate reader (Spark®; Tecan Group Ltd. Männedorf, Switzerland). The intra- and inter-assay coefficients of variation (CV) for the assay were 1.9% and 10.0%, respectively. 

Total protein concentration was measured using the Pierce^TM^ BCA Protein Assay Kit (Thermo Fisher Scientific, Scoresby Vic, Australia) after dilution of standards and samples in 2% sodium dodecyl sulfate (SDS; Invitrogen Life Technologies, Thermo Fisher Scientific, Scoresby Vic, Australia) to remove any lipid interference, as described by Geale [21]. Intra- and inter-assay CVs were 2.3% and 5.8%, respectively. Total IgG concentration was determined using a commercial ELISA kit with assays performed in singlicate (Bethyl Laboratories, Montgomery TX, USA; 1.6% inter-assay CV), and total lactose concentration was analyzed using a commercial colorimetric assay kit with assays performed in singlicate (BioVision; Sapphire Bioscience, Redfern NSW, Australia; 6.7% inter-assay CV).

### 2.6. Serum Metabolite Analysis

Serum beta-hydroxybutyrate (β-HBA) concentrations were assayed using a commercial colorimetric assay kit (Cayman Chemical; Sapphire Bioscience, Redfern NSW, Australia) according to the manufacturer’s instructions with an intra-assay CV of 2.1% and an inter-assay CV of 16.5%. Total serum glucose concentration was determined using a colorimetric assay (Infinity^TM^ Glucose Reagent, Thermo Fisher Scientific, Scoresby Vic, Australia) and a calibrator with a reference value of 177 mg/dL glucose as a standard (Data Cal^TM^, Thermo Fisher Scientific, Scoresby Vic, Australia), and the absorbance was read at 490 nm. Intra- and inter-assay CVs for the glucose assay were 2.2% and 14.3%, respectively. Serum non-esterified FA (NEFA) concentrations were determined using the NEFA C kit (Wako Pure Chemical Industries, Kawagoe, Japan) as per the manufacturer’s instructions, and intra- and inter-assay CVs were 2.7% and 1.9%, respectively. Total IgG and protein concentrations in each piglet serum sample were determined using the commercial kits described previously (protein assay intra-assay CV 2.6%; inter-assay CVs 12.6% and 4.6%, respectively). Serum samples from all female progeny in the CON and MCFA dietary treatment groups (*n* = 36) were analyzed for volatile FA (VFA) and FA methyl ester (FAME) concentrations at the NSW Department of Primary Industries (Wagga Wagga NSW, Australia) via gas-liquid chromatography according to the methods described by the AOAC [20].

### 2.7. Statistical Analysis

Growth and reproductive performance of dams, colostrum and milk composition, litter growth performance (with the dam as the experimental unit), and serum IgG and metabolite concentrations (with an individual piglet as the experimental unit) were analyzed as linear mixed models using the MIXED procedure of SPSS (IBM SPSS, version 24; IBM, Armonk NY, USA). Dam parity (primiparous versus multiparous), diet, and the interaction between the two were used as fixed factors in a 2 × 4 factorial design with other factors and covariates included in the model as necessary. A 2 × 2 factorial design was employed for the analysis of piglet serum FA concentrations (primiparous versus multiparous; CON versus MCFA). For the analysis of piglet serum IgG and metabolite concentrations, sex was fitted as a fixed factor where it made a significant contribution to the model. The number of days from day 107 of gestation to farrowing or to weaning was fitted as a covariate where they made a significant contribution to the model. This was in order to represent the number of days that the experimental diets were fed to the dam before farrowing, which was unbalanced in the study design due to commercial constraints and because farrowing did not always occur on the expected date. Pairwise comparisons between interaction means were made with simple effects analysis within the COMPARE function using SPSS syntax. Mortality data and subsequent mating and farrow rate were analyzed as binomial traits (e.g., lived or died, mated or not, farrowed or not) using chi square (χ^2^) in the CROSSTABS procedure of SPSS, and post hoc analysis was performed by comparing column proportions (individual treatment groups) using the *z* test. *P-*values of *p* < 0.05 were considered significant, and *p* < 0.10 were considered a trend.

## 3. Results

The number of primiparous and multiparous sows on each diet at the start of the experimental period is shown in Table 3. During the lactation period, four primiparous and four multiparous sows were removed from the experiment due to mortality or ill health. Of those sows, any that farrowed prior to removal had their farrowing house entry (BW and P2 backfat depth) and farrowing data (NBA, SB, MU, TB) included in the analysis. Seven primiparous and eight multiparous sows were removed for management and health reasons after weaning; hence, subsequent mating data were not available for these animals. The results of the analysis were similar whether fostered piglets were included or not. Therefore, the results presented herein include the results for these piglets. Piglet sex was not significant (*p* ≥ 0.05) for any trait and was therefore excluded from all statistical models. The number of days from day 107 of gestation to farrowing was used as a covariate in the analysis of dam P2 and BW at weaning, subsequent gestation length, piglet serum IgG concentration, litter weight at day 21, and NW in the experimental litter, as it made a significant (*p* < 0.05) contribution to the model, and the number of days from entry to weaning was used as a covariate in the analysis of dam BW change in lactation.

### 3.1. Dam Reproductive Performance

Dam BW at entry to the farrowing house and at weaning was lower (*p* < 0.001) in primiparous compared to multiparous sows (entry: 193.9 ± 1.8 vs. 281.5 ± 1.8 kg, respectively; weaning: 180.5 ± 1.9 vs. 256.6 ± 1.9 kg, respectively) and primiparous sows lost less (*p* < 0.001) BW from entry to weaning compared to multiparous sows (Table 4).

Multiparous sows on the CON diet were fatter at entry to the farrowing house than multiparous sows on other diets, whereas primiparous sows on the BOTH diet were fatter compared to primiparous sows on the other diets (*p* = 0.003; Table 4). Therefore, P2 backfat depth at entry nested within dam parity treatment was tested as a covariate in the analysis for any further parameters. However, it was not significant for any trait (*p* ≥ 0.10) and was therefore not included in any models except for that of the change in P2 backfat depth from entry to weaning. Dam P2 backfat depth at entry was different (*p* = 0.024) with respect to diet, with primiparous and multiparous sows in the CON treatment fatter than those in the experimental treatments (Table 4). There was no difference (*p* = 0.26) in P2 backfat depth at weaning between dietary treatments. The dam parity x diet interaction was significant (*p* = 0.004) for change in P2 backfat depth from entry to weaning (Table 4). However, when the change in P2 backfat depth from entry to weaning was corrected for P2 backfat depth at entry to the farrowing house (nested within dam parity treatment), there was no difference (*p* ≥ 0.05) between dam parities, diets, or their interaction (data not shown).

**Figure 1:**
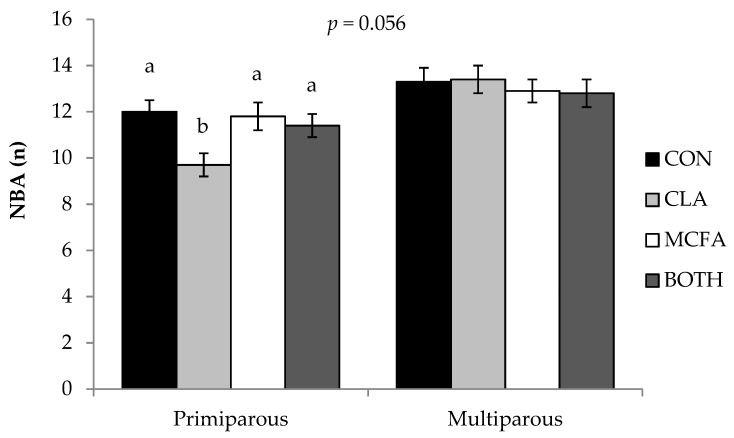
Treatment comparisons in number of piglets born alive (NBA) showing the interaction between dam parity (primiparous versus multiparous) and diet. ^ab^ Different superscripts denote values within dam parity group that are significantly different (*p <* 0.05).

The dam parity x diet interaction tended (*p* = 0.056) to influence the NBA (Figure 1). Primiparous sows fed the CLA diet had lower (*p* = 0.002) NBA than primiparous sows on the other diets, whereas multiparous sows from all dietary treatments had similar NBA (*p* ≥ 0.10). There was no effect (*p* ≥ 0.10) of dam parity, diet, or their interaction on subsequent WEI, remating, or farrowing rate, and there was no effect of diet or the dam parity x diet interaction (*p* ≥ 0.10) on any subsequent reproductive parameters (Table 4).

### 3.2. Colostrum and Milk Composition

Neither colostrum nor milk composition were affected (*p* ≥ 0.10) by dietary treatment, and there were no dam parity x diet interactive effects (*p ≥* 0.10; Table 5). Colostrum from primiparous sows tended to have a higher (*p* = 0.071) concentration of IgG and had higher fat (*p* = 0.007) and NE (*p* = 0.012) concentrations than colostrum from multiparous sows (Table 5), but there were no differences (*p ≥* 0.10) in milk. There were no other differences between primiparous and multiparous sows in any other colostrum or milk composition parameters measured (Table 5). 

### 3.3. Litter Growth Performance

**Figure 2:**
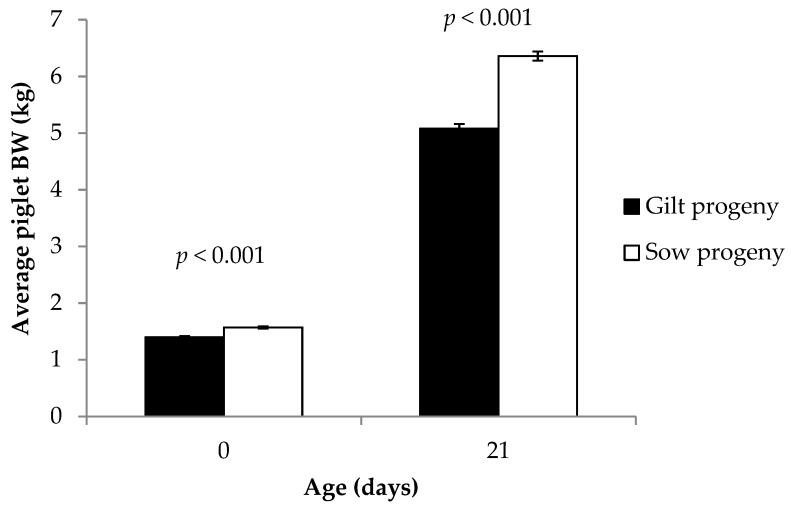
Effect of dam parity (primiparous versus multiparous; gilt versus sow progeny) on average piglet body weight (BW) at 0 and 21 days of age.

The dam parity x diet interaction was not significant (*p ≥* 0.10) for any litter growth performance traits (data not shown). Diet had no effect (*p ≥* 0.10) on total litter weight at birth, after standardization (fostering), or on day 21 of lactation (data not shown). Primiparous sow litters were significantly (*p <* 0.001) lighter than multiparous sow litters at all time points (birth: 15.1 ± 0.4 vs. 19.6 ± 0.4 kg, respectively; after standardization: 15.8 ± 0.3 vs. 18.6 ± 0.3 kg, respectively; day 21: 49.5 ± 1.2 vs. 64.8 ± 1.2 kg, respectively). There was no effect of diet on average piglet BW at birth (*p* = 0.84) or day 21 (*p* = 0.53), nor any average piglet BW gain from birth to day 21 (*p* = 0.47; data not shown). Gilt progeny were lighter than sow progeny in terms of average piglet BW at birth and at day 21 (*p* < 0.001; Figure 2) and gained less BW between birth and 21 days of age (3.71 ± 0.07 vs. 4.79 ± 0.08 kg, respectively; *p <* 0.001). 

**Figure 3:**
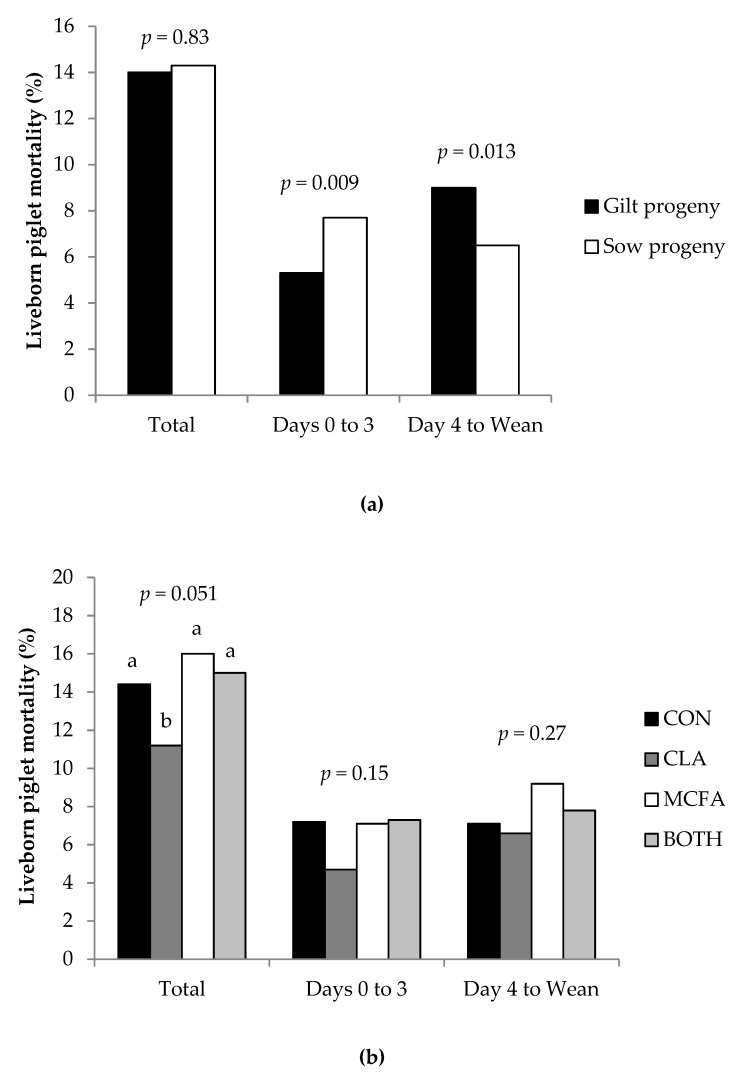
Effect of (**a**) dam parity (primiparous versus multiparous; gilt versus sow progeny) and (**b**) late gestation and lactation diet on pre-weaning liveborn piglet mortality. ^ab^ Different superscripts denote values that are significantly different (*p <* 0.05).

There was no difference (*p* = 0.83) in total liveborn pre-weaning mortality between gilt and sow progeny (Figure 3a). However, a higher proportion (*p* = 0.009) of sow progeny died in the first three days of life than gilt progeny, the effect of which was reversed in the remaining days to weaning (*p* = 0.013; Figure 3a). Piglets from primiparous and multiparous sows on the CLA diet tended to have the lowest (*p* = 0.051) liveborn pre-weaning mortality rate compared to those from the other diets (Figure 3b). There was no significant difference in liveborn pre-weaning mortality when divided into the first three days (*p* = 0.15) and the remaining days (*p* = 0.27) of lactation. 

### 3.4. Piglet Serum IgG and Metabolites

**Figure 4:**
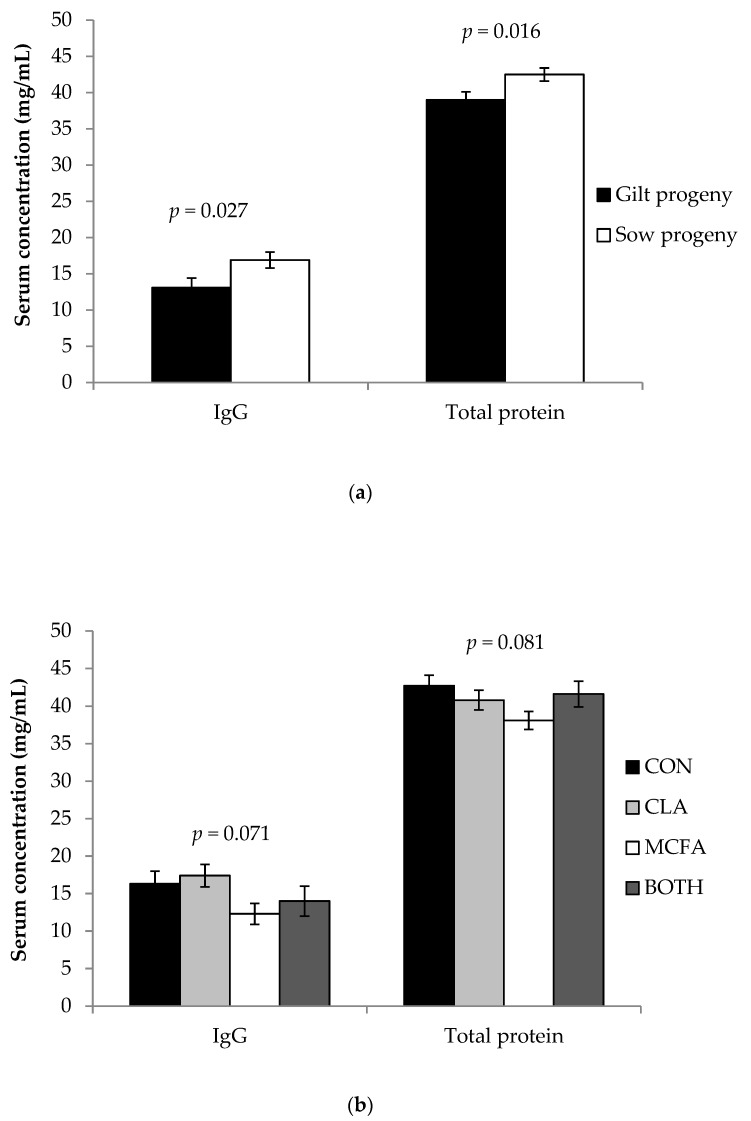
Effect of: (**a**) dam parity (primiparous versus multiparous; gilt versus sow progeny); and (**b**) late gestation and lactation on piglet serum immunoglobulin G (IgG) and total protein concentrations on day 3 of lactation.

The dam parity x diet interaction was not significant for piglet serum IgG or protein concentration (*p ≥* 0.05; data not shown). Gilt progeny had a lower serum concentration of IgG (*p* = 0.027) and protein (*p* = 0.016) than sow progeny on day three of lactation (Figure 4a). Diet tended to have an effect on serum IgG (*p* = 0.071) and protein concentration (*p* = 0.081) of piglets (Figure 4b). There were no dam parity, diet, or interactive effects on serum glucose, βHBA, NEFA, or TG concentrations of piglets (*p ≥* 0.10; Table 6). Apart from the tendency for piglets of primiparous and multiparous sows fed the MCFA diet to have higher (*p* = 0.083) serum concentrations of acetic acid than those from primiparous and multiparous sows on the CON diet, there were no other dam parity, diet, or interactive differences (*p ≥* 0.10) in any concentrations of the other serum FA (Table 7).

## 4. Discussion

The current study confirmed that gilt progeny are born lighter and grow slower before weaning compared to progeny born to multiparous sows [13,15,22]. Gilt progeny had lower serum IgG concentrations than sow progeny in the current study, as expected from previous findings [15,22]. However, colostrum and milk IgG concentrations did not differ between primiparous and multiparous sows in the current study. In fact, primiparous sows tended to have higher IgG concentrations in colostrum, a result in contention with previous work [23,24]. Differences between studies may be due to variation in a number of factors, such as timing of colostrum collection [25], udder section sampled [26], and multiparous sow parities included in the study [27], as colostrum IgG concentrations have been shown to be very sensitive to such differences in collection methodology. Furthermore, there may be differences in colostrum [25,28] and milk volume [29,30,31] between primiparous and multiparous sows, which may impact the concentrations of several components, including IgG.

Conjugated linoleic acid has been shown to have positive immunomodulatory effects on colostrum, milk, and progeny serum IgG concentrations when fed in late gestation and (or) lactation to sows [4,8,32], and MCFA have been shown to have antibacterial properties and improve gut development and function in the pig [11,33,34]. Despite these purported effects, we observed no added benefits to the lactation performance of primiparous sows and their progeny relative to sows and their progeny with CLA or MCFA. Overall, there were only subtle differences between the performance and serum IgG concentration of gilt and sow progeny when their dams were fed the experimental diets. Given the lack of improvements in performance between piglets from primiparous and multiparous sows fed CLA and MCFA separately, it was therefore not surprising that there were no advantages to feeding both ingredients together in the BOTH treatment. It was unfortunate that other immunoglobulins such as IgA and IgM were not measured in the current study, as feeding CLA from day 85 of gestation until farrowing has been shown to improve concentrations of IgA and IgM in sow serum, colostrum, and piglet serum [10]. Furthermore, dietary MCFA may have immunomodulatory effects, such as stimulating IgA production in response to a bacterial lipopolysaccharide (LPS) challenge [11]. Hence, these mechanisms require further investigation. The microbiome of the sow and (or) the piglets may also have been altered by feeding MCFA, which have been shown to have several probiotic and antibacterial effects in the intestinal lumen of pigs [11]. These may have improved progeny health and growth performance after weaning but were unable to be quantified in the current study.

Feeding CLA to dams during late gestation and lactation did not increase day three serum IgG concentrations of piglets in the present study. Similar studies that found an improvement in serum IgG of piglets found these differences at weaning [8,35] or after weaning [32]. Piglet IgG concentrations were not measured at or after weaning in the current study, and as such, it is unclear whether improvements in immune status may have been gained later in life. Liu et al. [36] found an improvement in progeny serum IgG concentration in the first 24 to 26 hours of life, but this was only in piglets from the highest CLA inclusion level treatment (2.25% CLA oil). They also found an increase in colostrum IgG concentrations at only the higher inclusion levels (> 1.5% CLA), whereas we found no difference at 0.5% CLA. This suggests that higher levels of CLA (> 0.5%) may be necessary in order to improve IgG concentrations in primiparous and multiparous sow colostrum and increase piglet serum IgG in the first postnatal days. 

Data as to whether CLA increases [37] or depresses [10] colostrum fat concentration are equivocal. Results from the current study concur with Cordero et al. [6] that there was no effect on colostrum fat concentration, which is also consistent with the studies of Bontempo et al. [4] and Schmid et al. [38] that found no effect on milk fat concentration. Other studies report that milk fat was depressed when dams were fed CLA [35,39,40,41]. Piglet growth performance was not affected by CLA supplementation in agreement with the previous literature [4,7]. However, Wu et al. [10] showed that piglet growth performance increased linearly in line with an increase in CLA inclusion level (up to 2.25% CLA oil; total 1.35% CLA isomers), which may indicate that the levels used in the current study were insufficient to noticeably impact piglet performance. This is further evidenced by the observation of increased milk yield and piglet pre-weaning growth rates in studies that fed 1% CLA oil (approximately 0.6% biologically active CLA isomers) or higher [6,35,37]. This may also be an explanation as to why there were no differences in piglet serum metabolites such as glucose or triglycerides from dams fed CLA in the current study.

In agreement with Hadaš et al. [42] and Liu et al. [36], liveborn mortality in the current study tended to be lower for piglets born to dams fed the CLA diet. However, Lee et al. [35] found no effect, and Barowicz et al. [43] and Krogh et al. [37] actually found higher mortality rates in piglets born to sows fed CLA. The finding that SB was higher in CLA-fed sows compared to CON sows—and that NBA was lower in CLA-fed primiparous sows than those primiparous sows on the other dietary treatments—was surprising, as most previous studies found no difference [8,42], and some even suggested that CLA increased NBA [5,43]. In addition, Liu et al. [36] found that feeding CLA had no effect on farrowing duration in primiparous sows, which suggests that increased farrowing difficulty was not the reason for higher SB or lower NBA in the current study. These differences may be due to inconsistencies between methodology, as there are variations between these studies in time period (e.g., first week, 21, 28, or 35 day lactations), inclusion level of CLA (ranging from 1 to 2.25% CLA product), and (or) average parity of the experimental sows (e.g., primiparous sows only, parities 2-5, 1-3, or 3-4). These studies also had a comparatively low number of animals studied compared to the present study (eight to twelve versus >30 dams per treatment), an important consideration when measuring variables such as pre-weaning mortality rates. Regardless, it seems from this study that liveborn pre-weaning mortality rates are improved by the inclusion of CLA in the sow diet for reasons other than improving colostrum and milk IgG concentration or piglet energy metabolism, as suggested by other authors who fed CLA at higher inclusion levels [4,5,6,8,10]. It is more likely that overall colostrum intake was increased in these piglets, and that the concentrations of CLA isomers in colostrum and milk were enhanced [4,6]. This may have had impacts on adipocyte function, protein accretion, hepatic lipid metabolism, and immune function [44] that may not have manifested as differences in serum metabolite concentrations of piglets at three days of age in the current study. It may therefore be more beneficial to feed CLA at these inclusion rates from farrowing until weaning rather than from day 107 of gestation, as was the case in the current study. This feeding regime may therefore have less of an impact on fetal development, the farrowing process, and hence the rate of stillbirths, but this requires further investigation.

Medium-chain FA can be found in their triglyceride form (MCT) in coconut oil, palm kernel oil, and cuphea seeds, or manufactured in their free FA form into commercial feed additives [11]. The antimicrobial effects of MCFA in the intestinal lumen of pigs have been well reported [45]. Mechanisms may include anionic surfactant function [34], destabilization of bacterial cell walls and membranes by incorporation into the membrane itself and (or) inhibition of bacterial lipase [46,47,48], initiating cell death via disassociating within the bacterial cell and lowering pH [49,50], or by triggering cell autolysis by activation of bacterial enzymes [51]. Dietary MCTs have also been shown to stimulate growth hormone (GH) release in pigs, most likely through increased bioactivation of ghrelin [52]. Moreover, due to their positive effects on energy homeostasis and gut health [53,54], MCFA may be effective in improving FA metabolism in sows, increasing milk FA content and suckling piglet energy levels as a result [55,56,57]. Our study showed no positive effects of feeding MCFA on progeny performance or serum metabolite profiles, suggesting that the inclusion level in the current study was insufficient to influence the piglet when fed through the dam. The fact that there was no increase in piglet pre-weaning growth performance when their dams were fed the MCFA diet is consistent with previous work in cows [57], and is in agreement with the lack of difference in CON and MCFA piglet energetic metabolite profiles. However, when fed as MCT, these FA are known to have a ketogenic effect on the sow and improve blood glucose metabolism [58], serum FA profiles [59], pre-weaning weight gain of her progeny [55], and pre-weaning survival of light birth weight piglets [60,61]. It is more likely (in this case) that the ketones produced by the dam as a result of the breakdown of MCTs are passed to the progeny via the placenta [11,62]. Ingredients with high levels of MCTs, such as coconut oil or palm kernel oil, may therefore be a more viable dietary alternative (compared to free FA products) to improve primiparous and multiparous sow and progeny performance. Feeding these additives in late gestation and (or) lactation has been shown to increase pre-weaning survival rates in low birth weight pigs [60,61,63]. However, the use of these products in a commercial setting may be limited by their cost and difficulties with mixing at the feed mill owing to their relatively high viscosity. This has also previously been identified as an issue with CLA oil addition [64].

## 5. Conclusions

In conclusion, feeding CLA or MCFA alone or in combination to primiparous and multiparous sows from day 107 of gestation to weaning did not improve colostrum or milk composition, nor did it improve the serum energetic metabolite profile or pre-weaning growth of their piglets. These results suggest that supplementing 0.5% CLA isomers may increase pre-weaning liveborn survival of both gilt and sow progeny while also increasing the number of SB piglets. There were no added benefits of these additives to gilt progeny or to feeding both CLA and MCFA simultaneously. Feeding CLA may be more successful in improving the performance and pre-weaning survival of gilt progeny if fed only from farrowing until weaning, and MCFA may have impacts on intestinal microbiota and hence progeny growth and survival after weaning. Both of these require further investigation.

## Figures and Tables

**Table 1:** Composition of experimental diets fed in late gestation and lactation.

	Diet ^1^		
Diet Composition	CON	CLA	MCFA
Ingredient (%)			
Wheat	52.6	52.6	52.6
Barley	20	20	20
Canola meal 36%	10	10	10
Soybean meal 47%	2.5	2.5	2.5
Meat meal 60%	5	5	5
Fish oil	0.4	0.4	0.4
Tallow	6	3.5	5.9
Conjugated linoleic acid ^2^		2.5	
Medium-chain fatty acids ^3^			0.1
Betaine	0.4	0.4	0.4
Limestone	1.1	1.1	1.1
Magnesium sulfate	0.4	0.4	0.4
Potassium chloride	0.2	0.2	0.2
Salt	0.4	0.4	0.4
Lysine	0.4	0.4	0.4
Threonine	0.1	0.1	0.1
DL-Methionine	0.02	0.02	0.02
Vitamin blend ^4^	0.13	0.13	0.13
Mineral blend ^5^	0.25	0.25	0.25
Antioxidants ^6^	0.02	0.02	0.02
Feed acidifier ^7^	0.3	0.3	0.3
Enzyme ^8^	0.05	0.05	0.05
Calculated composition (DM basis) ^9^			
Energy (MJ DE/kg)	14.8	14.8	14.7
Crude protein (%)	17.0	17.0	17.0
Crude fat (%)	8.0	8.0	7.9
Crude fiber (%)	3.8	3.8	3.8
Ash (%)	5.6	5.6	5.6
Available SID lysine (%)	0.85	0.85	0.85

CON = control diet; CLA = conjugated linoleic acid diet; DE = digestible energy; DM = dry matter; MCFA = medium-chain fatty acid diet; SID = standardized ileal digestible.

^1^ Diets were fed from day 107 of gestation (107.3 ± 0.1 days; mean ± SE) until weaning (27.2 ± 0.1 days of lactation). A fourth diet, BOTH, was fed during the experiment which consisted of 50% CLA diet and 50% MCFA by weight.

^2^ Commercial 20% CLA product (Lutrell® Pure, BASF Australia, Southbank, Victoria) consisting of 10% c9,t11-18:2 and 10% t10,c12-18:2.

^3^ Commercial MCFA product (Aromabiotic® Pig, Nuscience, Drongen, Belgium) consisting of 28.1% acetic acid, 0.9% valeric acid, 0.9% caproic acid, 0.4% caprylic acid, 18.5% capric acid, 16.5% lauric acid (DM basis).

^4^ Supplied per kg of diet: vitamin A, 15000 IU; vitamin D3, 3200 IU; vitamin K, 1 mg; vitamin B-1, 1.5 mg; vitamin B-2, 5 mg; vitamin B-6, 3 mg; vitamin B-12, 135 µg; niacin, 20 mg; pantothenic acid, 15 mg, folic acid, 20 mg; vitamin C, 100 mg; biotin, 200 µg; Vitamin E, 90 mg.

^5^ Supplied per kg of diet: Cu 10 mg; Mn 40 mg; Zn 70 mg; Fe 100 mg; I 2.5 mg; Se 0.4 mg; Cr 500 μg, as organic trace minerals.

^6^ Endox^TM^ Dry (Kemin Industries, Des Moines, Iowa, USA).

^7^ Fysal® Feed (Trouw Nutrition, Nutreco, Amersfoort, Netherlands).

^8^ Rovabio® Max (Adisseo, Antony, France).

^9^ All diets had identical calculated compositions of Ca, P, and essential amino acids.

**Table 2:** Analyzed composition (dry matter basis) of experimental diets fed in late gestation and lactation.

Chemical Composition	Diet		
	CON	CLA	MCFA
Crude protein (%)	16.4	16.3	17.0
Crude fat (%)	8.8	8.1	9.4
Moisture (%)	9.5	9.7	9.3
Fatty acids (g/100 g total fatty acids)			
C8:0	ND	ND	0.31
C9:0	ND	0.03	ND
C10:0	ND	0.30	0.82
C11:0	0.06	0.07	0.06
C12:0	0.13	0.14	0.29
C16:0	16.6	21.1	23.5
C16:1n-7	1.7	1.8	2.4
C18:0	8.2	19.8	13.4
C18:1n-9	35.0	25.5	28.7
C18:2n-6	21.1	16.7	16.3
C18:3n-3	3.6	1.7	1.8
c9,t11 CLA	0.1	1.1	0.1
t10,c12 CLA	ND	1.0	0.1
ΣSFA	28.6	45.5	42.8
ΣMUFA	43.3	29.6	33.5

CON = control diet; CLA = conjugated linoleic acid diet; MCFA = medium-chain fatty acid diet; ND = not detected; ΣSFA = sum of total saturated fatty acids; ΣMUFA = sum of total monounsaturated fatty acids.

**Table 3:** Number of primiparous and multiparous sows on each experimental diet. Diets included a control diet (CON), a diet supplemented with conjugated linoleic acid (CLA), a medium-chain fatty acid (MCFA) product, or a combination of both CLA and MCFA (BOTH).

Dam Parity	Number of Sows (*n*)			
	Diet			
	CON	CLA	MCFA	BOTH
Primiparous	31	33	30	31
Multiparous	30	30	33	30

**Table 4:** Current and subsequent reproductive performance of primiparous and multiparous sows when fed either a commercial control diet (CON), a diet supplemented with conjugated linoleic acid (CLA), a medium-chain fatty acid (MCFA) product, or a combination of both CLA and MCFA (BOTH) in late gestation and lactation.

	Dam Parity (P)		Diet (D)				*p-*value		
Parameter	Primiparous	Multiparous	CON	CLA	MCFA	BOTH	P	D	P x D
Lactation performance									
ΔBW (kg)	−13.6 ± 1.3	−25.2 ± 1.3	−23.2 ± 1.9	−17.5 ± 1.9	−19.6 ± 1.8	−17.4 ± 1.9	< 0.001	0.10	0.60
P2_E_ (mm)	20.5 ± 0.4	28.0 ± 0.4	25.6 ± 0.6 ^a^	23.3 ± 0.6 ^b^	23.4 ± 0.6 ^b^	24.9 ± 0.6 ^ab^	< 0.001	0.024	0.003
P2_W_ (mm)	19.4 ± 0.4	24.2 ± 0.4	22.4 ± 0.6	21.2 ± 0.6	21.1 ± 0.6	22.4 ± 0.6	< 0.001	0.26	0.25
ΔP2 (mm)	−1.3 ± 0.3	−4.0 ± 0.3	−3.6 ± 0.4	−2.0 ± 0.4	−2.6 ± 0.4	−2.5 ± 0.4	< 0.001	0.061	0.004
ADFI (kg/d)	5.2 ± 0.1	5.9 ± 0.1	5.6 ± 0.1	5.6 ± 0.1	5.7 ± 0.1	5.4 ± 0.1	< 0.001	0.50	0.30
Litter performance									
NBA (n)	11.2 ± 0.3	13.1 ± 0.3	12.6 ± 0.4	11.6 ± 0.4	12.3 ± 0.4	12.1 ± 0.4	< 0.001	0.25	0.056
SB (n)	0.8 ± 0.1	0.9 ± 0.1	0.5 ± 0.1 ^a^	1.2 ± 0.1 ^b^	0.8 ± 0.1 ^a^	0.8 ± 0.1 ^a^	0.59	0.005	0.48
MU (n)	0.1 ± 0.1	0.3 ± 0.1	0.2 ± 0.1	0.3 ± 0.1	0.3 ± 0.1	0.1 ± 0.1	0.039	0.23	0.45
TB (n)	12.1 ± 0.3	14.3 ± 0.3	13.4 ± 0.4	13.1 ± 0.4	13.4 ± 0.1	12.9 ± 0.4	< 0.001	0.80	0.15
NW (n)	9.7 ± 0.2	10.2 ± 0.2	9.9 ± 0.2	10.1 ± 0.2	9.9 ± 0.2	9.9 ± 0.3	0.039	0.93	0.74
Subsequent reproductive performance									
Remated (%)	91.2	90.2	88.5	92.1	90.5	91.8	0.80	0.90	-
WEI (days)	5.7 ± 0.4	4.8 ± 0.5	4.4 ± 0.6	5.2 ± 0.6	6.5 ± 0.6	4.9 ± 0.6	0.12	0.16	0.36
FR (%)	74.6	82.0	79.6	72.4	77.2	83.9	0.18	0.51	-
GL (days)	115.8 ± 0.1	115.5 ± 0.1	115.5 ± 0.1	115.7 ± 0.1	115.6 ± 0.1	115.8 ± 0.1	0.055	0.43	0.17
NBA (n)	12.1 ± 0.3	13.3 ± 0.3	12.9 ± 0.4	12.7 ± 0.4	13.0 ± 0.4	12.2 ± 0.4	0.005	0.63	0.29
NW (n)	10.0 ± 0.3	9.4 ± 0.2	9.6 ± 0.3	10.1 ± 0.4	9.7 ± 0.4	9.4 ± 0.3	0.069	0.52	0.98

ADFI = average daily feed intake; ΔBW = change in body weight from farrowing house entry to weaning; ΔP2 = change in P2 backfat depth from farrowing house entry to weaning; FR = farrowing rate; GL = gestation length; MU = number of mummified fetuses; NBA = number born alive; NW = number weaned; P2_E_ = P2 backfat depth at entry to the farrowing house; P2_W_ = P2 backfat depth at weaning; SB = number of stillbirths; TB = total born; WEI = wean-to-estrus interval.

^a,b^ Values within a row with different superscripts differ significantly at *p <* 0.05.

**Table 5:** Colostrum and milk composition of primiparous and multiparous sows when fed either a commercial control diet (CON), a diet supplemented with conjugated linoleic acid (CLA), a medium-chain fatty acid (MCFA) product or a combination of both CLA and MCFA diets (BOTH) in late gestation and lactation.

	Dam Parity (P)		Diet (D)				*p*-value ^1^	
Parameter	Primiparous	Multiparous	CON	CLA	MCFA	BOTH	P	D
Colostrum (day 0)								
NE (MJ/kg)	3.5 ± 0.2	2.7 ± 0.2	3.1 ± 0.3	3.0 ± 0.3	3.2 ± 0.3	3.1 ± 0.4	0.012	0.96
IgG (mg/mL)	93.1 ± 9.5	70.9 ± 7.5	82.8 ± 12.7	81.0 ± 10.4	85.5 ± 10.7	78.8 ± 14.2	0.071	0.98
Fat (%)	7.8 ± 0.6	5.6 ± 0.5	6.7 ± 0.8	6.3 ± 0.7	6.9 ± 0.7	6.6 ± 0.9	0.007	0.94
Protein (%)	17.5 ± 0.5	16.8 ± 0.4	18.0 ± 0.7	16.3 ± 0.6	17.0 ± 0.6	17.4 ± 0.8	0.31	0.28
Lactose (%)	2.9 ± 0.1	3.1 ± 0.1	2.8 ± 0.2	3.2 ± 0.2	3.0 ± 0.2	3.0 ± 0.2	0.15	0.51
Milk (day 21)								
NE (MJ/kg)	4.0 ± 0.3	3.8 ± 0.2	4.2 ± 0.3	3.9 ± 0.3	3.6 ± 0.4	3.8 ± 0.4	0.53	0.66
IgG (mg/mL)	0.36 ± 0.04	0.31 ± 0.04	0.34 ± 0.06	0.30 ± 0.05	0.37 ± 0.05	0.33 ± 0.06	0.36	0.80
Fat (%)	5.9 ± 0.6	5.3 ± 0.5	6.3 ± 0.8	5.7 ± 0.6	4.7 ± 0.8	5.7 ± 0.9	0.44	0.61
Protein (%)	2.0 ± 0.2	2.0 ± 0.2	2.1 ± 0.3	1.7 ± 0.2	2.2 ± 0.3	1.8 ± 0.3	0.90	0.35
Lactose (%)	6.8 ± 0.2	7.1 ± 0.2	7.0 ± 0.3	6.8 ± 0.2	6.8 ± 0.3	7.1 ± 0.3	0.31	0.83

^1^ The interaction (dam parity x diet) was not significant (*p ≥* 0.10) for any trait.

**Table 6:** Serum metabolite concentrations of piglets on day 3 of lactation after dams fed either a commercial control diet (CON), a diet supplemented with conjugated linoleic acid (CLA), a medium-chain fatty acid (MCFA) product or a combination of both CLA and MCFA diets (BOTH) in late gestation and lactation.

	Diet				*p-*value
Parameter	CON	CLA	MCFA	BOTH	Diet
Serum concentration (mmol/L)					
GLUC	8.76 ± 0.17	8.57 ± 0.16	8.59 ± 0.15	8.50 ± 0.21	0.77
βHBA	0.81 ± 0.08	0.81 ± 0.07	0.82 ± 0.07	0.81 ± 0.09	1.00
NEFA	0.73 ± 0.08	0.62 ± 0.08	0.64 ± 0.07	0.72 ± 0.10	0.77
TG	1.55 ± 0.13	1.84 ± 0.11	1.67 ± 0.10	1.71 ± 0.14	0.39

GLUC = glucose; βHBA = beta-hydroxybutyrate; NEFA = non-esterified fatty acids; TG = triglycerides.

**Table 7:** Piglet serum fatty acid concentrations on day 3 of lactation after dams fed either a commercial control diet (CON), or a diet supplemented with a medium-chain fatty acid (MCFA) product in late gestation and lactation.

	Diet ^1^		*p-*value
Parameter	CON	MCFA	Diet
Serum concentration (μg/mL)			
Acetic acid	266 ± 5	278 ± 5	0.083
Butyric acid	14.0 ± 0.5	14.1 ± 0.4	0.86
Valeric acid ^2^	1.5 ± 0.5	0.9 ± 0.4	0.33
Caproic acid	3.5 ± 0.3	3.2 ± 0.2	0.45
Caprylic acid ^2^	0.7 ± 0.2	0.9 ± 0.2	0.80
Pelargonic acid ^2^	1.3 ± 0.3	1.6 ± 0.3	0.50
Capric acid ^2^	2.0 ± 0.4	1.6 ± 0.4	0.68
Undecyclic acid	1.7 ± 0.2	2.0 ± 0.2	0.31
Lauric acid	6.7 ± 0.5	7.4 ± 0.4	0.68

^1^ Results are based on a sub-sample of female piglets (*n* = 36). ^2^ Some pigs had serum concentration recorded as < 0.10 μg/mL. These have been assumed as zero for the analysis.
